# Supplementary material for: Prevention of Cardiovascular Diseases with Standard-Dose Quadrivalent Influenza Vaccine in People Aged ≥50 Years in Australia During the 2017 A/H3N2 Epidemic
Source: Vaccines (Basel). 2025 Apr 14;13(4):407. doi: 10.3390/vaccines13040407 (PMC12030789; doi:10.3390/vaccines13040407)
Supplement: Supplementary file 1 [file vaccines-13-00407-s001.zip › vaccines-3521430-supplementary.pdf]

Supplementary Materials:

Scheme S1. reports the aORs for all unmeasured confounders. Each of the unmeasured risk factor must have aORs greater than 12.82 to have any residual confounding on the effect of influenza vaccination on cardiovascular admission.

Risk Factors	Adjusted Odds Ratio, OR (95% CI)	P value
University of Higher Level of Education	3.08 (2.64 to 3.58)	<0.001
Private Insurance	1.52 (1.40 to 1.67)	<0.001
Consuming CVD medications	1.41 (1.29 to 1.54)	<0.001
